# Differential glucose uptake response to IGF-II and vesiculin in insulin-resistant muscle and adipose cells

**DOI:** 10.1042/BSR20250378

**Published:** 2026-05-13

**Authors:** Thai Nguyen, Caren Leemberg, Kate L. Lee

**Affiliations:** 1 School of Medical Sciences, Faculty of Medical and Health Sciences, University of Auckland, Auckland, New Zealand; 2 Institute for Life Sciences & Chemistry, Hogeschool Utrecht, Utrecht, Netherlands; 3 Maurice Wilkins Centre for Molecular Biodiscovery, Auckland, New Zealand

**Keywords:** adipocytes, insulin resistance, insulin-like growth factor, muscle metabolism

## Abstract

Type 2 diabetes (T2D), a significant global health concern, is characterised by insulin resistance and progressive pancreatic β-cell degradation leading to failure of blood glucose regulation. Pancreatic islet hormones are essential for systemic glucose homeostasis, and their dysregulation is implicated in T2D pathogenesis. Characterisation of these hormones has been critical for understanding disease progression and designing therapeutic approaches. Vesiculin is a two-chain peptide derived from insulin-like growth factor-II (IGF-II) via a four-amino-acid excision. Studies of IGF-II and vesiculin revealed comparable insulin-like glucoregulatory activity in mice; however, in insulin-resistant animal models, vesiculin activity remains intact, whereas IGF-II activity is impeded, highlighting potential pharmacological relevance for vesiculin.

We aimed to compare the glucoregulatory activity of vesiculin and IGF-II in differentiated 3T3-L1 adipocytes and L6-GLUT4myc myoblasts under insulin-sensitive and insulin-resistant conditions to understand tissue-specific activities of these peptides. Further, we characterised binding efficiency of vesiculin and IGF-II to several IGF-binding proteins (IGFBPs).

Vesiculin exhibited glucose uptake comparable to IGF-II in both cell types, with similar dose response curves. Vesiculin retained efficacy in insulin-resistant myotubes, whereas IGF-II activity was blunted; however, both peptides were not significantly impacted by insulin resistance in adipocytes. Vesiculin also showed a modest synergistic effect with insulin in resistant adipocytes. Additionally, binding assays revealed that vesiculin had significantly lower affinity for IGFBP3 and IGFBP5 compared with IGF-II. These findings demonstrate that vesiculin exhibits a distinct glucoregulatory signalling profile from IGF-II, discernible specifically under insulin-resistant conditions despite their structural similarity.

## Introduction

Type 2 diabetes (T2D) is a growing health issue worldwide, with nearly 590 million cases in 2024 and projected to increase by 45% worldwide by 2050 [1]. The two features of T2D are insulin resistance and progressive degradation of the pancreatic β-cells, the latter being critical for eventual reliance on therapeutic insulin. The disease is a multifactorial, heritable condition with both genetic and environmental factors involved [2]. Therefore, a ‘one size fits all’ drug regimen is often ineffective at keeping insulin dependency at bay in the long term. To mitigate this, new drugs will be required tackling the problem from different angles, with the aim of better targeting therapeutic options to the underlying drivers of disease which differ between individuals [3]. New drug discoveries have often come from understanding the hormones involved in glucose metabolism [4,5]. Hormones secreted by the pancreas are crucial for the maintenance of normal whole-body glucose homeostasis. Genetic variants that lead to the development of T2D are often associated with dysregulation in islet hormone expression and activity [6]. It is therefore important and beneficial to understand the roles of novel islet hormones.

Islet β-cells have long been known to contain an IGF-II-immunoreactive component [7], and further exploration of this revealed a two-chain peptide derived from mature insulin-like growth factor-II (IGF-II) that was named vesiculin [8]. After developing mass spectrometry-based assays that are highly specific, vesiculin was found to be secreted in response to glucose along with mature IGF-II [9]. Vesiculin has been shown to be bioactive and has equal potency to IGF-II in terms of glucoregulatory activity *in vivo* [10].

As part of a comparative exploration of the activity of vesiculin versus mature IGF-II, peptide tolerance tests revealed blood glucose-lowering activity of vesiculin was maintained in insulin-resistant mouse models, whereas IGF-II activity was hampered [10]. Therefore, we aimed to further explore the glucoregulatory effects of vesiculin and IGF-II in insulin-sensitive and insulin-resistant cell models of the two key tissues responsible for glucose clearance from circulation in response to insulin: fat and muscle. As our previous comparative data on activity in insulin-resistant states were in whole animals [10], we wanted to look in tissue-specific models to assess if differences in activity also occurred in these cells in isolation or if the differences we saw in whole animals were due to another factor, such as, differential interaction with circulating factors. We also aimed to further explore whether there were differences between vesiculin and IGF-II in terms of peptide interaction with IGF-binding proteins (IGFBPs).

## Results

### The glucose uptake activity of vesiculin is comparable to that of IGF-II in insulin-sensitive cells

**Figure 1:**
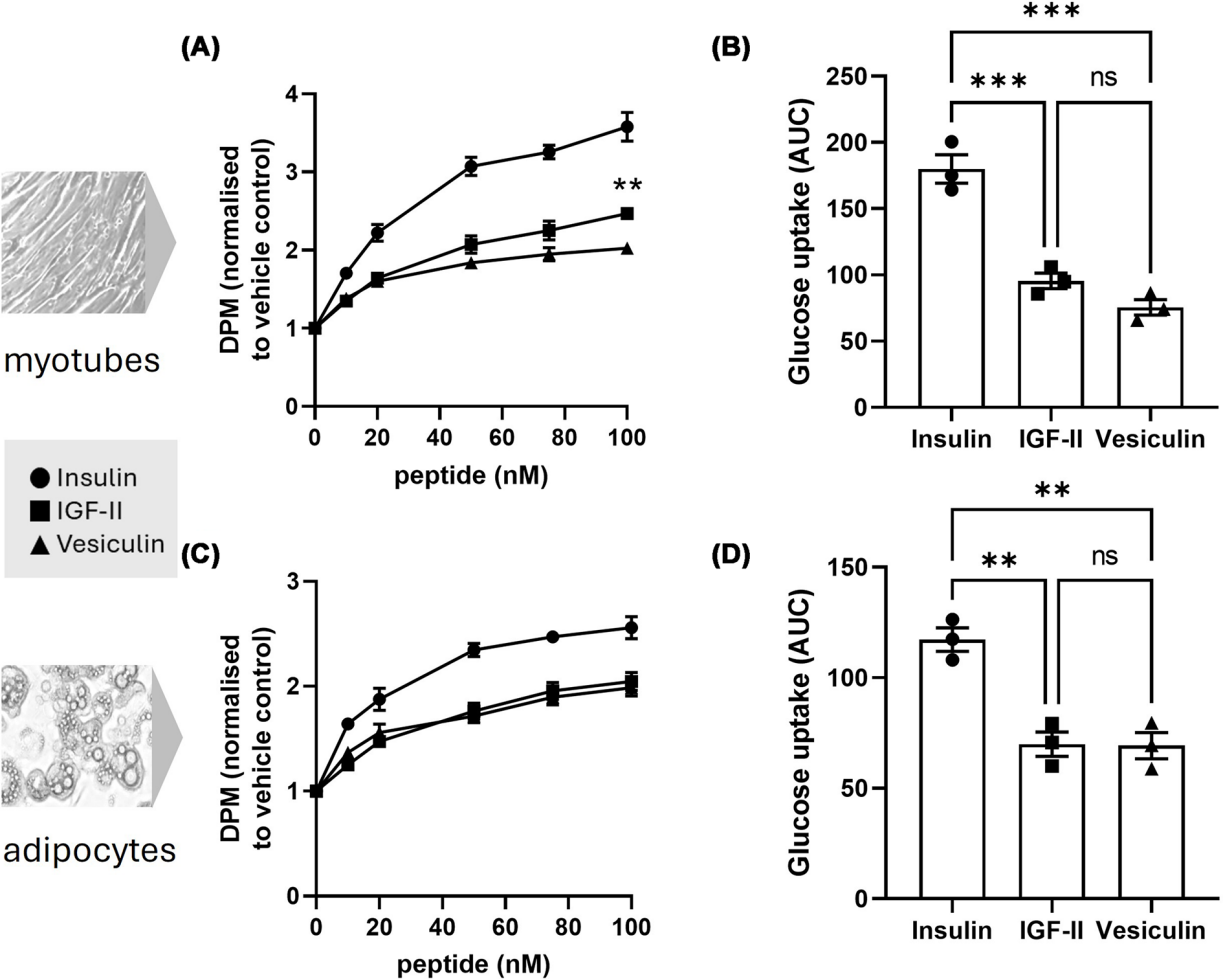
Glucose uptake in myotubes (A, B) and adipocytes (C, D) stimulated by insulin, IGF-II and vesiculin Dose-response curve of glucose uptake in response to insulin, IGF-II and vesiculin as measured by change of DPM (disintegrations per minute) relative to vehicle control for 3 independent experiments (mean ± SEM) in myotubes (**A**) and in adipocytes (**C**). Difference between IGF-II and vesiculin action at each concentration was analysed using FDR-corrected multiple t-test, using two-stage step-up (Benjamini, Krieger, and Yekutieli) method (A,C). AUC of evoked glucose uptake by insulin, IGF-II and vesiculin in myotubes (**B**) and adipocytes (**D**), calculated from dose-response curve data shown in panels A and C respectively. Statistical analysis on AUC data (B,D) was analysed using one-way ANOVA with mixed-effects analysis followed by Tukey multiple comparison test. Significant differences between each pair are labelled as follows: **P value ≤0.01, ***P value ≤0.001.

Dose response curves (up to 100 nM peptide) of glucose uptake elicited by all three peptides in differentiated L6 myotubes were generated (Figure 1A). Under this experimental model, insulin was able to elicit a stronger response than both IGF-II peptides, with an average maximal 3.6-fold change increase in glucose uptake (95% CI 2.8–4.4) over no-peptide control values with an EC50 of approximately 22.4 nM. IGF-II was able to elicit 2.47-fold increase (CI 2.2–2.8) with an EC50 of 26.5 nM, whereas vesiculin elicited 2.0-fold increase (CI 1.7–2.3) with an EC50 of 16 nM. Multiple t-tests comparing IGF-II and vesiculin revealed a significant difference in response at 100 nM, with IGF-II having a significantly higher maximal response (Figure 1A). Comparing the area under the curve (AUC) for all three dose-response curves revealed activity of insulin is approximately double that of the IGF-II peptides (180 versus 95 for IGF-II and 75 for vesiculin), and there was a small, but not statistically significant difference between the responses elicited by IGF-II and vesiculin (Figure 1B). As shown in Figure 1C, the relative glucose uptake activity of insulin in adipocytes elicited a larger maximal response (mean 2.6-fold change, CI 2.1–3.0, EC50 16 nM) than both IGF-II (2.0-fold change, CI 1.7–2.4, EC50 25 nM) and vesiculin (2.0-fold change, CI 1.7–2.3, EC50 17 nM). AUC revealed insulin to have approximately double activity of IGF-II and vesiculin (117 versus 70 for IGF-II and 69 for vesiculin) with no significant difference between IGF-II and vesiculin (Figure 1D).

### Vesiculin retains its effect in insulin resistance

**Figure 2:**
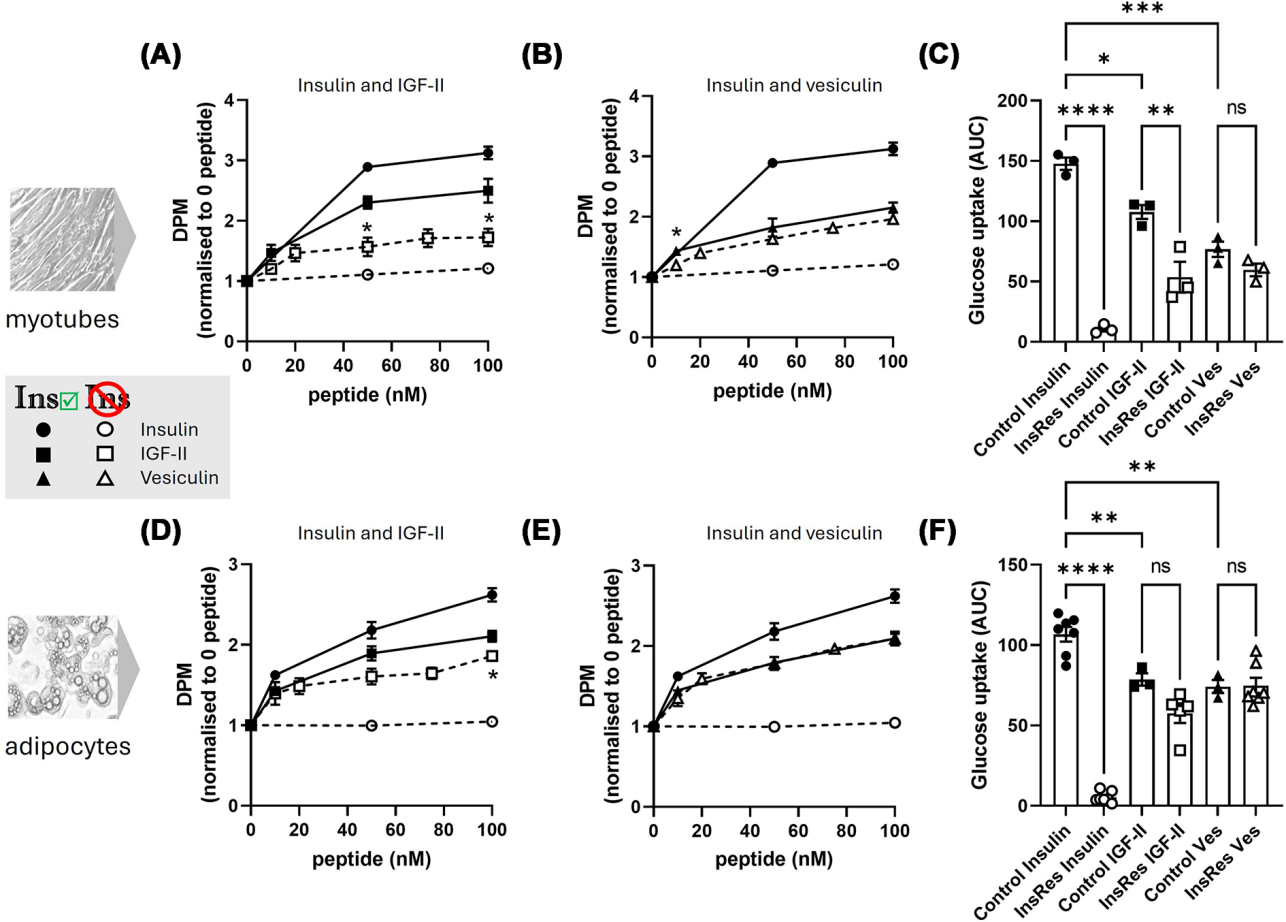
Glucose uptake in insulin-resistant and insulin-sensitive myotubes (A–C) and adipocytes (D–F) Dose-response curves for insulin and IGF-II (**A,D**) and insulin and vesiculin (**B,E**). Note: IGF-II and vesiculin are shown in separate panels with insulin as positive and negative controls to make data more visible; insulin data shown in panels (A,B) as well as (D,E) is the same data. Data from insulin-sensitive cells are shown by closed symbols and data from insulin-resistant cells are shown as open symbols as per key in figure. The data are measured by change of DPM relative to vehicle control for 3 independent experiments (mean ± SEM) (A, B, D, E). Changes in peptide action (at each concentration) in insulin-resistant versus insulin-sensitive cells were analysed using FDR-corrected multiple t-test, using two-stage step-up (Benjamini, Krieger, and Yekutieli) method (A, B, D, E). AUC of evoked glucose uptake in insulin-sensitive (closed symbols) and insulin-resistant (open symbols) myotubes (**C**) and adipocytes (**F**) calculated from dose-response curves shown in panels (A,B and D,E) respectively. AUC data were analysed using one-way ANOVA and Šídák’s multiple comparisons test. Significant differences between each pair are labelled as follows: *P value ≤0.05, **P ≤0.01, ***P ≤0.001 and ****P <0.0001.

Treatment to induce insulin resistance in muscle cells was effective. In palmitate-treated L6 myotubes, insulin exerted very little stimulation of glucose uptake, with its maximal response approximately 20% above that of unstimulated control (Figure 2A–C). IGF-II activity was also drastically reduced in insulin-resistant L6 myotubes with a maximum fold change of 1.7 (CI 1.1–2.3) versus 2.5-fold change (CI 1.7–3.3) in insulin-sensitive myotubes (Figure 2A). Multiple t-tests at 10, 50, and 100 nM doses comparing IGF-II response in insulin-sensitive versus insulin-resistant cells revealed a significant difference at both 50 and 100 nM (Figure 2A). Maximal fold change increase in glucose uptake induced by vesiculin in palmitate-treated cells was 2.0 (CI 1.7–2.3) versus 2.2-fold change (CI 1.8–2.5) in non-treated, insulin-sensitive cells (Figure 2B). Multiple testing of vesiculin response at 10, 50, and 100 nM did reveal a statistical difference at 10 nM, but no difference at 50 or 100 nM. Comparing AUC for the peptides under different conditions revealed significant difference between IGF-II response in insulin-sensitive versus insulin-resistant cells (AUC 108 and 54, respectively, *P* = 0.002), whereas the difference in vesiculin activity was not statistically significant (AUC 77 and 60, respectively, *P* = 0.8) (Figure 2C).

Dexamethasone-treated differentiated adipocytes displayed complete resistance to insulin (Figure 2D–F). In insulin-resistant adipocytes, IGF-II activity was slightly reduced with maximum fold change 1.9 (CI 1.7–2.0) in insulin-resistant versus 2.1 (CI 1.8–2.4) in insulin-sensitive cells (Figure 2D). Multiple t-tests of IGF-II response between sensitive versus resistant cells at 10, 50, and 100 nM indicated a significantly lower response at 100 nM (Figure 2D). Vesiculin response was almost identical in both conditions, with maximum response of 2.1 (CI 1.9–2.2) in insulin-resistant versus 2.1 (1.7–2.5) in insulin-sensitive cells (Figure 2E). There was a small difference in AUC for IGF-II activity between insulin-resistant vs insulin-sensitive (58 versus 79, respectively), although this didn’t reach statistical significance. AUC for vesiculin between insulin-resistant versus insulin-sensitive (75 versus 74, respectively) was not different.

Overall, the insulin-resistant state had a more marked impact on IGF-II than vesiculin, particularly in L6 muscle cells.

### Both peptides drive glucose uptake via PI3k/Akt, but only vesiculin can synergise with insulin

**Figure 3:**
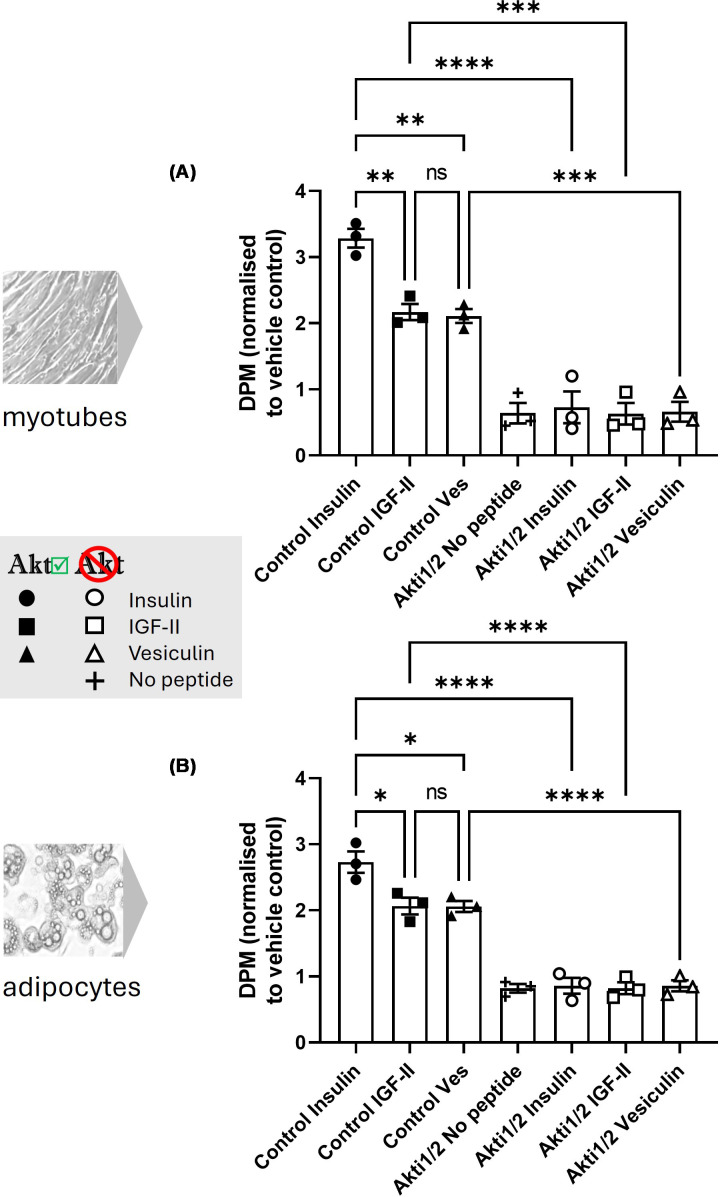
Glucose uptake in control (vehicle) versus Akti1/2-treated myocytes (A) and adipocytes (B) stimulated by 100 nM of insulin, IGF-II or vesiculin Uptake in control vehicle-treated cells is shown in closed symbols, and Akti1/2-treated cells are shown in open symbols. (**A**,**B**) The data are measured by change of DPM relative to vehicle control for 3 independent experiments (mean ± SEM). (A,B) One-way ANOVA and Šídák’s multiple comparisons test were carried out to compare the effect of Akt inhibition on the action of each peptide. Significant differences between each pair are labelled as follows: *P value ≤0.05, **P ≤0.01, ***P ≤0.001 and ****P <0.0001.

The activity of both IGF-II and vesiculin has been shown to be through the same family of highly conserved receptors, including the insulin receptor (IR) and the insulin-like growth factor 1 receptor (IGF1R) [10,11]. Activation of these receptors results in anabolic metabolic responses as well as growth and proliferation. Insulin-sensitive cells were treated with Akti-1/2 to selectively and efficiently inhibit Akt isoforms 1 and 2. Glucose uptake assays were then performed to investigate whether Pi3k/Akt is critical for the glucose uptake activity of both peptides. None of the peptides (including insulin) were able to evoke an increase in glucose uptake in Akti-1/2-treated L6 myotubes or adipocytes (Figure 3A,B, respectively).

**Figure 4:**
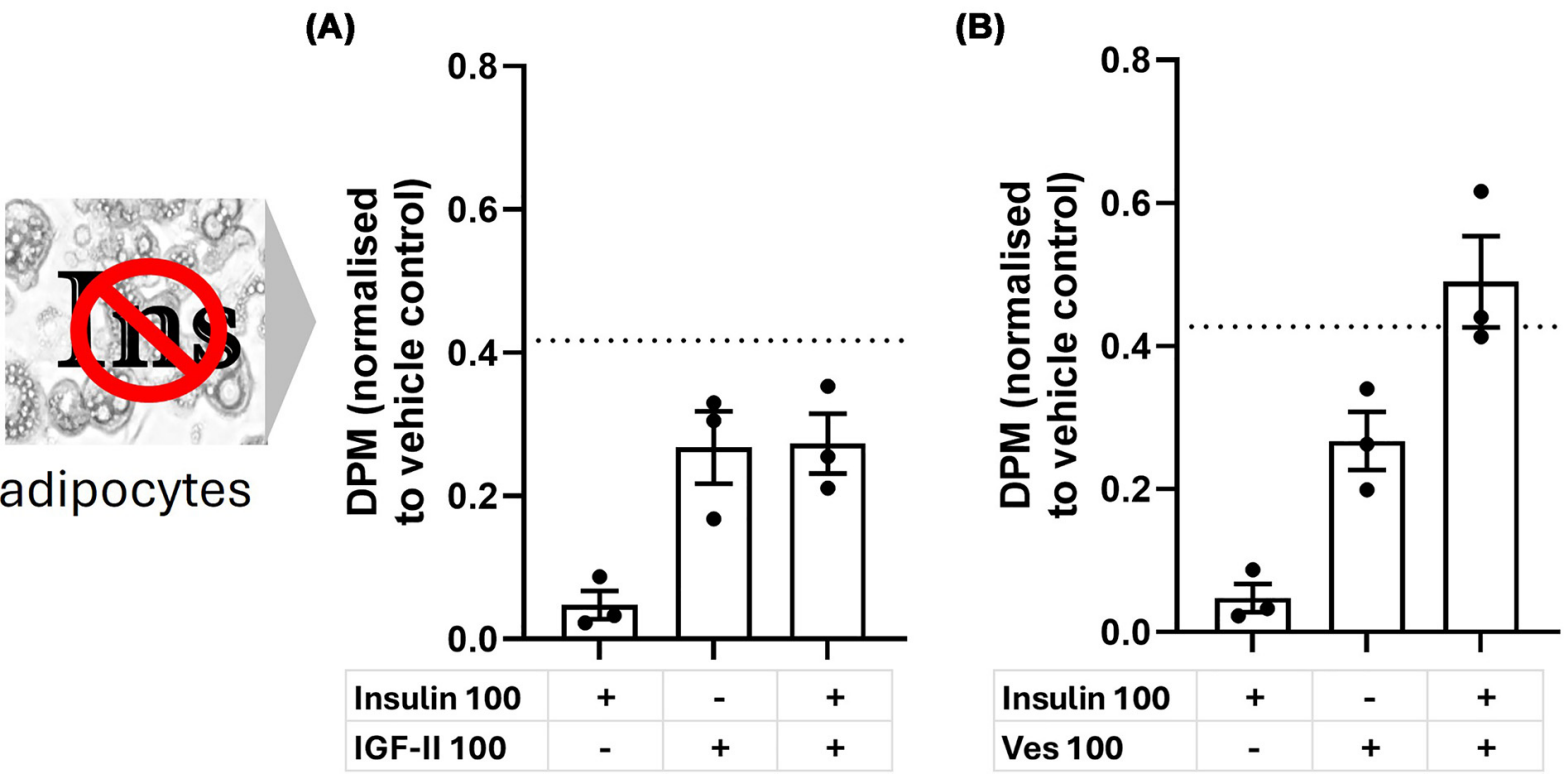
Additive versus synergistic effect of insulin and IGF-II (A) or insulin and vesiculin (B) in insulin-resistant adipocytes DPM normalised to no peptide vehicle from *n* = 3 independent glucose uptake experiments (mean ± SEM) in insulin-resistant adipocytes. Peptide concentrations were 100 nM (50 nM and 100 nM data are shown in Supplementary Table S1). Dotted line shows the ‘predicted additive’ response as estimated by using the maximum response of each peptide added together.

Insulin, IGF-I and IGF-II all have different affinities to the suite of receptors they signal through, and although both IGF-II and vesiculin signal through this same family (and use the Pi3k/Akt pathway to cause glucose uptake), there may be subtle differences in receptor interaction. As we showed differential activity in insulin-resistant 3T3-L1 adipocytes, we wanted to test whether either IGF-II or vesiculin had potential to act synergistically with insulin in these cells. Insulin-resistant 3T3-L1 adipocytes were treated with IGF-II or vesiculin alone or co-incubated with insulin at peptide concentrations of 50 and 100 nM (Figure 4 and Supplementary Table 1). The ‘predicted additive’ response was calculated from summing single peptide response data (mean, minimum and maximum). This was compared with the actual double peptide responses. Our definition for synergism was achieved if the mean of actual double peptide response was above the maximum value of the ‘predicted additive’ response. IGF-II was unable to elicit a synergistic response (Figure 4A). One hundred nanomolar of vesiculin was able to elicit a synergistic response with 100 nM insulin based on our criteria (Figure 4B). Further data on both 50 and 100 nM combinations of peptides is shown in Supplementary Table 1.

### IGF-II has a stronger affinity to insulin-like growth factor binding protein 3 than vesiculin

**Figure 5:**
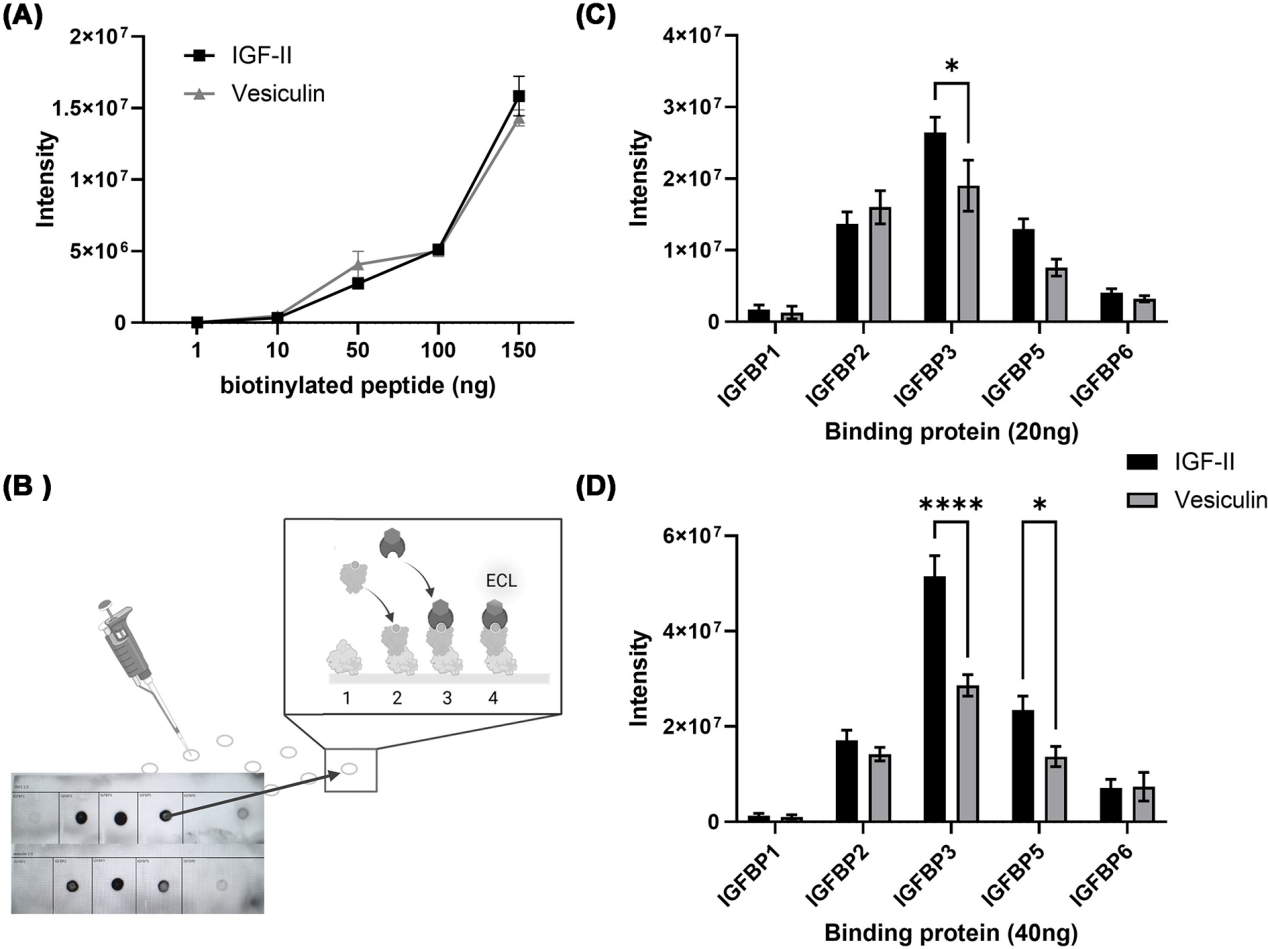
Binding of biotinylated IGF-II (C) or vesiculin (D) to immobilised IGFBPs Concentration series of biotinylated IGF-II and vesiculin immobilised on nitrocellulose membrane and detected using ECL reagent, *n* = 3 (**A**). Illustration of method, created in BioRender by Shepherd, P. (2026) https://BioRender.com/cwxjgdc: (1) recombinant IGFBPs immobilised on PVBF membrane, (2) incubated with biotinylated IGF-II or vesiculin, (3) incubated with Streptavidin-HRP, and (4) chemiluminescent detection (**B**). Densitometry results from either 20 µg (**C**) or 40 ng (**D**) of immobilised binding proteins incubated with either biotinylated IGF-II or vesiculin. The data were analysed using one-way ANOVA and Šídák’s multiple comparisons test. Significant differences between each pair are labelled as follows: *P value ≤0.05, and ****P <0.0001. Raw blots and quantitation available at https://doi.org/10.6084/m9.figshare.30880427.

In the first instance, we checked for differences in intensity of the labelling of our two biotinyated peptides using serially diluted labelled peptides and found no significant difference across a range of concentrations (Figure 5A). We then tested binding of biotinylated peptide to IGFBPs that were immobilised on PVDF membrane (method illustrated in Figure 5B). This assay detected a difference in binding affinity of our ligands to insulin-like growth factor binding protein 3 (IGFBP3) at 20 and 40ng, and at 40 ng there was also a difference in binding to IGFBP5. In all cases vesiculin had lower affinity to the IGFBPs (Figure 5C,D).

## Discussion

We focused our comparative analysis of IGF-II and vesiculin on *in vitro* models of muscle and fat and used these models under insulin-sensitive and insulin-resistant states to explore differences in IGF-II and vesiculin activity. Insulin elicited the highest maximal glucose uptake response in insulin-sensitive states in both cell models. IGF-II and vesiculin were also capable of inducing glucose uptake in muscle and adipose cells, though at a significantly lower efficacy than insulin, in agreement with previous *in vivo* and *ex vivo* data [10,12]. Vesiculin’s maximal activity in the myotubes was slightly lower than that of IGF-II but the effect of both peptides was identical in the adipocytes. Glucose uptake response to insulin was almost completely abolished in both cell lines following inducement of insulin resistance by well-characterised methods. Response to IGF-II was reduced in insulin-resistant muscle cells compared with insulin-sensitive cells. In contrast, vesiculin activity was not significantly altered by insulin resistance. Insulin-resistant adipose cells had a small reduction in IGF-II activity that wasn’t statistically significant, whereas again vesiculin activity was identical to that in insulin-sensitive cells. The result is consistent with findings from insulin-resistant mouse models [10].

We have shown in these cell models that vesiculin is indeed unimpeded by insulin resistance, whereas IGF-II activity is blunted, but this difference may be specific to muscle. As skeletal muscle has a larger impact on postprandial glucose disposal in response to insulin signalling [13], this difference in the activity of IGF-II versus vesiculin in insulin-resistant states would translate to the differences we saw in whole-animal studies [10]. This indicates that glucose-lowering signals from IGF-II in the muscle of insulin-resistant mouse models are being negatively impacted by some factor that does not impact vesiculin’s glucoregulatory activity. There is still debate over the molecular mechanisms of insulin resistance, although reduced expression and/or translocation of glucose transporters is a key mechanism. As the protocol we used for driving insulin resistance in the two cell types was different (lipotoxicity in L6 muscle cells versus dexamethasone in 3T3-L1 adipocytes), this may explain the different relative responses to our two peptides in the two cell lines. Further work will be needed to explore whether vesiculin signalling increases glucose transporter expression and translocation to the membrane in the face of lipotoxicity. Importantly though, as we see the differential response in muscle cells in culture, this suggests that a local factor rather than a secondary effect of peptide responses (i.e., on other organs or interactions with circulating factors) is impacting IGF-II and not vesiculin.

Peptide combinations were tested in insulin-resistant adipocytes. IGF-II showed no indication of synergism at the concentrations and combinations tested. Interestingly, there was also little evidence for additive activity. This phenomenon may be due to the competition between insulin and IGF-II to interact with the same group of receptors and thus further weaken the effect of both peptides. Vesiculin was able to elicit what we defined as a synergistic effect with insulin at the higher doses tested. Synergism suggests that in the state of insulin resistance, vesiculin may be able to enhance cell response to insulin stimulation to some degree. Furthermore, the difference in these data between IGF-II and vesiculin may indicate some subtle differences in receptor preference and binding affinities.

The ability of vesiculin to bypass insulin resistance when IGF-II signalling is blunted could be driven by several potential mechanisms. Previous data indicated that vesiculin, like IGF-II, acts through the insulin family of RTKs. The first consideration is that IGF-II and vesiculin have different preferences for the IR and IGF1R, although data from knockout cells suggested this isn’t the case [10]. However, there is yet no data to indicate if there is a difference in affinity for signalling via the A and B isoforms of the IR. A second consideration is that IGF-II and vesiculin have critical differences in downstream signalling pathways that account for these differences; however, use of Akt inhibitor fully ablated the ability of all peptide hormones to induce glucose uptake. This shows they all have the PI3K/Akt pathway as a critical component of their activity; however, the potential of more subtle biased signalling accounting for these differences has not been ruled out [14,15].

In humans, IGFs are present in the circulation at high levels, 1000× higher than insulin (whereas mice have vanishingly low circulating levels of IGF in adulthood). Bioactivity of circulating IGFs is limited by the presence of IGFBPs. Binding of IGFs to IGFBPs moves half-life from minutes to hours. IGFPBs are not only circulating but are increasingly understood to have local tissue expression. According to GTEx (accessed Jan 25), IGFBP-5 has predominant expression in female reproductive tissues, followed by skeletal muscle and adipose. IGFBP3 again has strong expression in some female reproductive tissues, with liver and adipose tissue also high; skeletal muscle expression of IGFBP3 seems relatively low. IGFBP3 is the most abundant IGFBP in circulation. Some IGFBPs are known to be associated with insulin resistance, and therefore differential affinity for binding proteins could be responsible for increased inhibition of IGF-II activity relative to vesiculin in our animal models. Indeed, we saw that both IGFBP3 and IGFBP5 had relatively lower binding affinity for vesiculin compared with IGF-II. Therefore, if either is expressed locally in myocytes and adipocytes and elevated in insulin-resistant states, these may be important for the differences in activity we see. There is a paucity of data indicating changes in levels of IGFBPs in insulin-resistant states, but both IGFBP3 and IGFBP5 have been linked to insulin resistance, poorer islet function, obesity, as well as future diabetes risk [16]. The metalloproteinase pappalysin 2 (PAPPA2) is responsible for cleavage of both IGFBP3 and IGFBP5, therefore driving IGF bioavailability. PAPPA2 genetic variants have been linked to hyperinsulinemia and insulin resistance, and the KO mouse model also has a metabolic phenotype but as they are also linked to growth retardation, the metabolic phenotypes could be secondary [17]. There is no data yet to characterise tissue-specific changes in PAPPA2 in insulin resistance, but our data suggests suppressed PAPPA2 activity could also be a candidate mechanism for suppressing IGF-II activity and sparing vesiculin activity. A final contender for a factor impacting IGF-II function in insulin-resistant states is the multifunctional protein IGF2R, also known as the cation-independent mannose-6-phosphate receptor. This receptor binds to and sequesters IGF-II for degradation and is generally more highly expressed in muscle than adipose tissues (GTEx, accessed Jan 25). IGF2R is known to be up-regulated in obesity and has strong genetic links to diabetes progression [18,19]. Further exploration of the local role of IGFBPs and IGF2R in regulating IGF-II and vesiculin activity in insulin resistance and diabetes is needed.

Vesiculin is likely to be specifically expressed in β-cells, but not in other cell types producing IGF-II due to the high concentrations of the necessary prohormone convertases in the secretory granule. It is yet to be established if vesiculin may be produced in other neuroendocrine cell types that also express prohormone convertases [20]. In pre-diabetic/insulin-resistant mice, pharmacological vesiculin was found to initiate islet cell proliferation [21], a role known to be linked to islet IGF-II expression [22–24]. Much of the data on vesiculin to date is based on glucoregulatory activity, which is a useful assay of peptide function, but as vesiculin is not thought to be in circulation [9] and is also unlikely to be produced locally in muscle or fat tissues, this data is limited in informing us of the natural role of vesiculin in β-cells. Although our data does suggest a potential utility for vesiculin as a diabetes therapeutic. Together, the results presented here and prior data from whole animals [10,21] suggest vesiculin has distinct activity from IGF-II and may be important for β-cell protection and proliferation in times of stress when IGF-II and insulin activity are diminished. More detailed analysis of the activity and mechanism of IGF-II and vesiculin in β-cell function, proliferation and as a cytoprotectant is needed. Both cell lines studied here were of male origin, so extrapolations to female biology, particularly premenopausal, is problematic, and future work should focus on replicating in females.

## Materials and methods

### Materials

Recombinant mouse IGF-II and recombinant mouse IGFBP1, 2, 3, 5, and 6 were from R&D Systems (Minneapolis, Minnesota, U.S.A.). IGF-II was reconstituted at a concentration of 1 g/l in sterile phosphate-buffered saline pH 7.4. Mouse vesiculin was synthesised in-house according to a published procedure [12]. Stocks were prepared at 1 μg/μl in 16 mg/ml glycerol. Actrapid^®^ recombinant human insulin 100 IU/ml, 3.47 g/l was from Novo Nordisk (Bagsvaerd, Denmark). EZ-Link Micro NHS-PEG_4_-Biotinylation Kit, Dulbecco’s modified Eagle’s media (DMEM), L-glutamine, newborn calf serum (NCS, NZ origin), horse serum (NZ origin) and antibiotic-antimycotic (Anti-Anti) were from Life Technologies (Thermo Scientific, Albany, New Zealand). Nitrocellulose Membrane, Clarity Western ECL Substrate, SDS and EDTA were from Bio-Rad. Foetal bovine serum (FBS, NZ origin) was from Moregate BioTech (Hamilton, New Zealand). Unless otherwise noted, all other chemicals were from Sigma–Aldrich (St Louis, MO).

### Cell culture

L6-GLUT4myc myoblasts originally derived from male rat (Kerafast, Boston, MA, U.S.A.) (Wang, Khayat, Kishi, Ebina & Klip, 1998), were used between passage 4 and 15 and were maintained in DMEM containing 1 g/l (5.55 mM) glucose, 10% (v/v) FBS, 1% (v/v) Ant-Anti, 1% (v/v) L-glutamine and 110 mg/l (1 mM) sodium pyruvate at 37°C and 5% CO_2_ atmosphere. Complete DMEM was replaced every 2 days and cells were split once they reached 80% confluence. L6 myoblasts at 100% confluency were induced to differentiate by treating with high-glucose (4.5 g/l or 25 mM) DMEM containing 2% horse serum, 1% Anti-Anti, 1% L-glutamine, and 1 mM sodium pyruvate. Temperature and CO_2_ levels were maintained, with media replaced every 2 days until mature myotubes formed.

3T3-L1 fibroblasts originally derived from male mice were used between passages 4–12 and were cultured in DMEM containing 4.5 g/l (25 mM) glucose with 10% NCS, 1% Anti-Anti, 1% L-glutamine, and 1 mM sodium pyruvate at 37°C and 5% CO_2_. Media was changed every 2 days, and cells were passaged before clumping. 3T3-L1 cell differentiation to adipocytes was initiated at 100% confluence. Differentiation media consisted of high-glucose DMEM with 10% FBS, 1% Anti-Anti, 1% L-glutamine, 1 mM sodium pyruvate, 0.5 mM IBMX, 0.1 μM dexamethasone, 100 nM insulin, and 10 nM troglitazone and was applied for 4 days. On day 5, the media was replaced with complete DMEM containing 100 nM insulin. After 2 days, fresh complete DMEM was added without additives, and media was changed every two days until >90% cells developed oil droplets.

Both L6 and 3T3-L1 cells were seeded to a 12-well plate at a density of 1.5 × 10^5^ cells per well for glucose uptake experiments. Insulin resistance was induced in fully differentiated myotubes by treating them with 200 μM palmitate for 16 h, while differentiated adipocytes were treated with 1 μM dexamethasone and 100 nM insulin for 24 h.

### Glucose Uptake

Differentiated L6-GLUT4myc cells were serum-starved in DMEM with 0.2% BSA for 3 h, then washed 3× with sterile HEPES-buffered saline (HBS; 140 mM NaCl, 20 mM HEPES, 2.5 mM MgSO_4_, 1 mM CaCl_2_, 5 mM KCl, pH 7.4, 25°C). Cells were first given a 10-min incubation in HBS containing 100 mM 2-deoxyglucose and 0.1% BSA, followed by a 20-min pre-stimulation with peptide. After that, cells were incubated in HBS containing [3H]-2-deoxyglucose, 0.2% BSA, and either vehicle controls or peptide treatment. After 10 min, glucose uptake was stopped by removing the media, placing cells on ice, and washing 5× with ice-cold PBS. Cells were lysed in 10% Triton X, then added to scintillation fluid, and absorption was measured using a beta counter (Tri-Carb 2910 TR Liquid Scintillation Analyzer). The same procedure was carried out for 3T3-L1 cells, but instead of HBS, Krebs–Ringer bicarbonate HEPES buffer (KRBH) was used.

### Akt inhibition

L6 myotubes and differentiated 3T3-L1 adipocytes were washed with PBS then incubated for 3 h in serum-free DMEM containing 2 mM glucose, 0.2% (w/v) fat-free BSA and 1 μM Akt kinase inhibitor (Akti 1/2) prior to performing a glucose uptake assay as above.

### Binding assays

Peptides were biotinylated following manufacturers’ instructions. Either 40 ng or 20 ng mouse IGFBP of each IGFBP (1, 2, 3, 5, and 6) was dotted (2 μl) onto a nitrocellulose membrane and dried at 37°C for 5 min. The blots were incubated at room temperature with agitation, first in TBS with 3% BSA for 2 h, then in TBS with 0.1% Tween 20 for 10 min. The blot was then incubated overnight with 40 ng of biotinylated peptides in TBS with 1% BSA and 0.1% tween at 4°C with agitation. The blots were then washed three times with TBS with 1% BSA and 0.1% tween 20 for 5 min at room temperature before being incubated for 1 h with Streptavidin-HPR (1:2000 dilution) in TBS with 1% BSA and 0.1% tween 20. Further wash steps preceded incubation with ECL reagent and imaging and densitometry analysis (Image Lab, BioRad). For testing equal signal from both biotinylated peptides, different concentrations of both labelled peptides were directly dotted on the membrane, which was dried, washed, incubated with Streptavidin-HPR, washed, and then detected.

### Statistics

For dose-response curve data, FDR-corrected multiple t-test using two-stage step-up procedure (Benjamini, Krieger, and Yekutieli) was used to compare the peptides at each concentration. We also took the overall response from our dose-response curve data and calculated an AUC for each individual experiment, and these were compared using one-way ANOVA with mixed-effects analysis followed by Tukey’s multiple comparison test or Šídák’s multiple comparison test where appropriate (indicated in figure legends). When comparing glucose uptake data from Akt inhibition data or chemiluminescent signal intensities One-way ANOVA with mixed-effects analysis followed by Šídák’s multiple comparison test was used.

## Supplementary Material

Supplementary Table S1

## Open Access

Open access for this article was enabled by the participation of the University of Auckland in an all-inclusive Read & Publish agreement with Portland Press and the Biochemical Society under an agreement with CAUL.

## Abbreviations

Anti-Anti: antibiotic-antimycotic
AUC: area under the curve
DMEM: Dulbecco’s modified Eagle’s medium
DPM: disintegrations per minute
FBS: Foetal bovine serum
IGF1R: insulin-like growth factor 1 receptor
IGF-II: insulin-like growth factor-II
IGF2R: Insulin-like growth factor 2 receptor
IGFBP3: insulin-like growth factor binding protein 3
IGFBP5: insulin-like growth factor binding protein 5
IGFBPs: IGF-binding proteins
IR: insulin receptor
PAPPA2: pappalysin 2
T2D: Type 2 diabetes
