# Supplementary material for: Differential glucose uptake response to IGF-II and vesiculin in insulin-resistant muscle and adipose cells
Source: Biosci Rep. 2026 May 13;46(5):BSR20250378. doi: 10.1042/BSR20250378 (PMC13185126; doi:10.1042/BSR20250378)
Supplement: Supplementary Table S1 [file BSR-2025-0378-T_supp.pdf]

A

Single Peptide Data	Ins 0	Ins 50	Ins 100
IGF-II 0		**0.07** (0.02 - 0.10)	**0.05** (0.02 - 0.09)
IGF-II 50	**0.18** (0.13 - 0.25)		
IGF-II 100	**0.27** (0.17 - 0.33)		
Predicted Additive	Ins 0	Ins 50	Ins 100
IGF-II 0			
IGF-II 50		**0.25** (0.15 - 0.36)	**0.22** (0.16 - 0.34)
IGF-II 100		**0.34** (0.19 - 0.43)	**0.32** (0.19 - 0.42)
Double Peptide Data	Ins 0	Ins 50	Ins 100
IGF-II 0			
IGF-II 50		**0.18** (0.08 - 0.27)	**0.27** (0.21 - 0.34)
IGF-II 100		**0.27** (0.21 - 0.32)	**0.27** (0.21 - 0.35)

B

Single Peptide Data	Ins 0	Ins 50	Ins 100
Ves 0		**0.07** (0.02 - 0.10)	**0.05** (0.02 - 0.09)
Ves 50	**0.21** (0.17 - 0.28)		
Ves 100	**0.27** (0.20 - 0.34)		
Predicted Additive	Ins 0	Ins 50	Ins 100
Ves 0			
Ves 50		**0.28** (0.19 - 0.39)	**0.26** (0.19 - 0.37)
Ves 100		**0.34** (0.22 - 0.44)	**0.31** (0.22 - 0.43)
Double Peptide Data	Ins 0	Ins 50	Ins 100
Ves 0			
Ves 50		**0.38** (0.32 - 0.50)	**0.42** (0.36 - 0.52) #
Ves 100		**0.37** (0.24 - 0.50)	**0.49** (0.41 - 0.62) #
